# Publisher Correction: ‘TRAIL-based gene delivery and therapeutic strategies’ and ‘Overview of recent advances in liposomal nanoparticle-based cancer immunotherapy’

**DOI:** 10.1038/s41401-020-0444-0

**Published:** 2020-07-06

**Authors:** Hui-ha Zhong, Hui-yuan Wang, Jian Gao, Yong-zhuo Huang, Ang Gao, Xian-li Hu, Madiha Saeed, Bin-fan Chen, Ya-ping Li, Hai-jun Yu

**Affiliations:** 1 Shanghai University College of Sciences, Sanghai, 200444 China; 2 State Key Laboratory of Drug Research, Shanghai Institute of Materia Medica, Chinese Academy of Sciences, Shanghai, 201203 China; 3 School of Pharmaceutical Science, Jiangsu University, Zhenjiang, 212013 China; 4 State Key Laboratory of Drug Research & Center of Pharmaceutics, Shanghai Institute of Materia Medica, Chinese Academy of Sciences, Shanghai, 201203 China; 5 School of Chemistry and Molecular Engineering, East China Normal University, Shanghai, 200241 China; 6 University of Chinese Academy of Sciences, Beijing, 100049 China

Correction to: *Acta Pharmacologica Sinica* 10.1038/s41401-019-0287-8, published online 23 August 2019; 10.1038/s41401-019-0281-1, published online 01 August 2019

The articles listed above were initially published with incorrect copyright information or changed later to incorrect copyright information.

Upon publication of this Correction, the copyright of these articles changed to “The Author(s)”.

The original articles have been corrected.

